# Supplementary material for: Association between passing return-to-sport testing and re-injury risk in patients after anterior cruciate ligament reconstruction surgery: a systematic review and meta-analysis
Source: PeerJ. 2024 Apr 29;12:e17279. doi: 10.7717/peerj.17279 (PMC11064852; doi:10.7717/peerj.17279)
Supplement: Supplemental Information 1 [file peerj-12-17279-s001.docx]

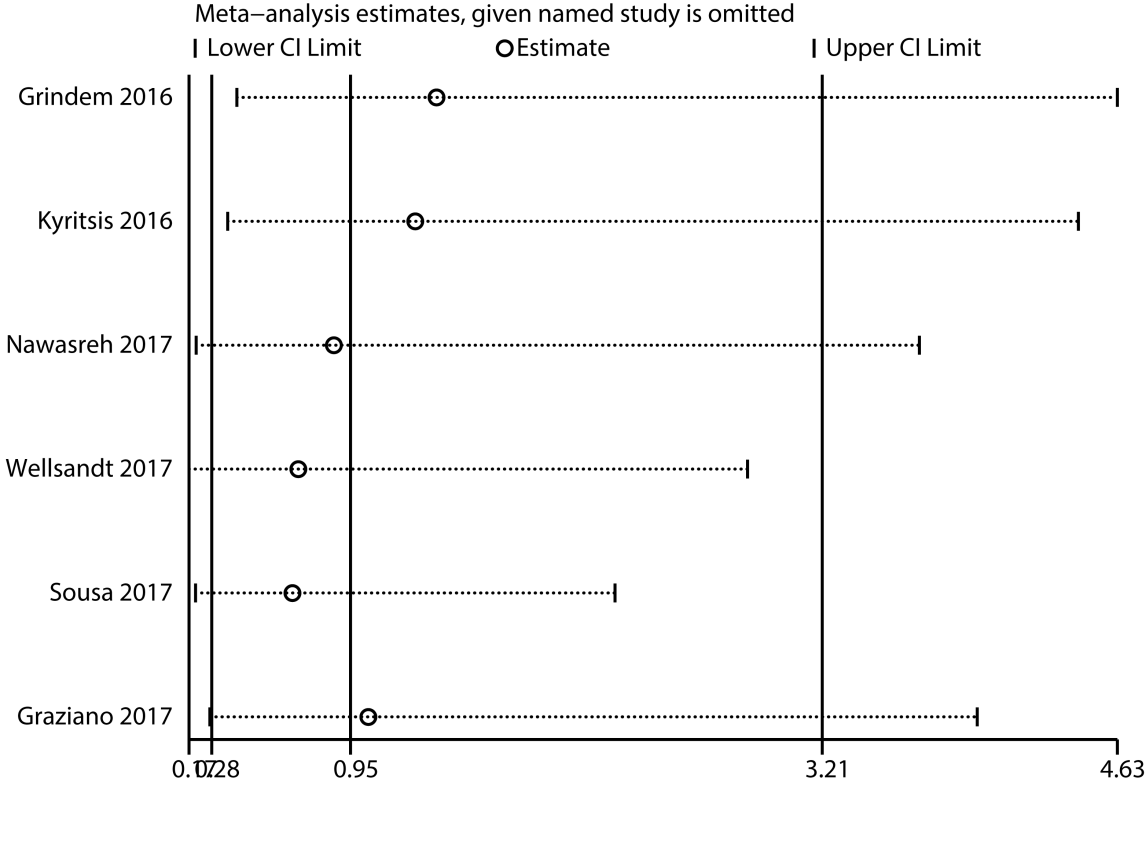


Figure S1. Sensitivity analysis for knee injury


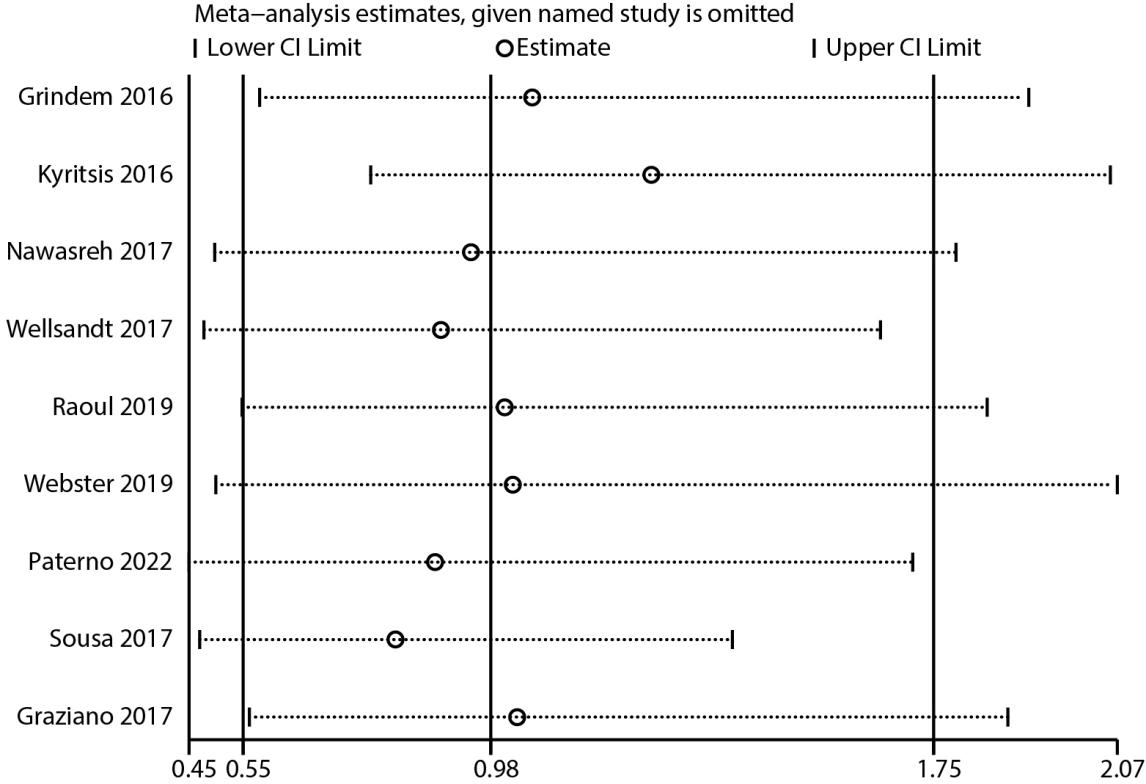


Figure S2. Sensitivity analysis for secondary ACL injury


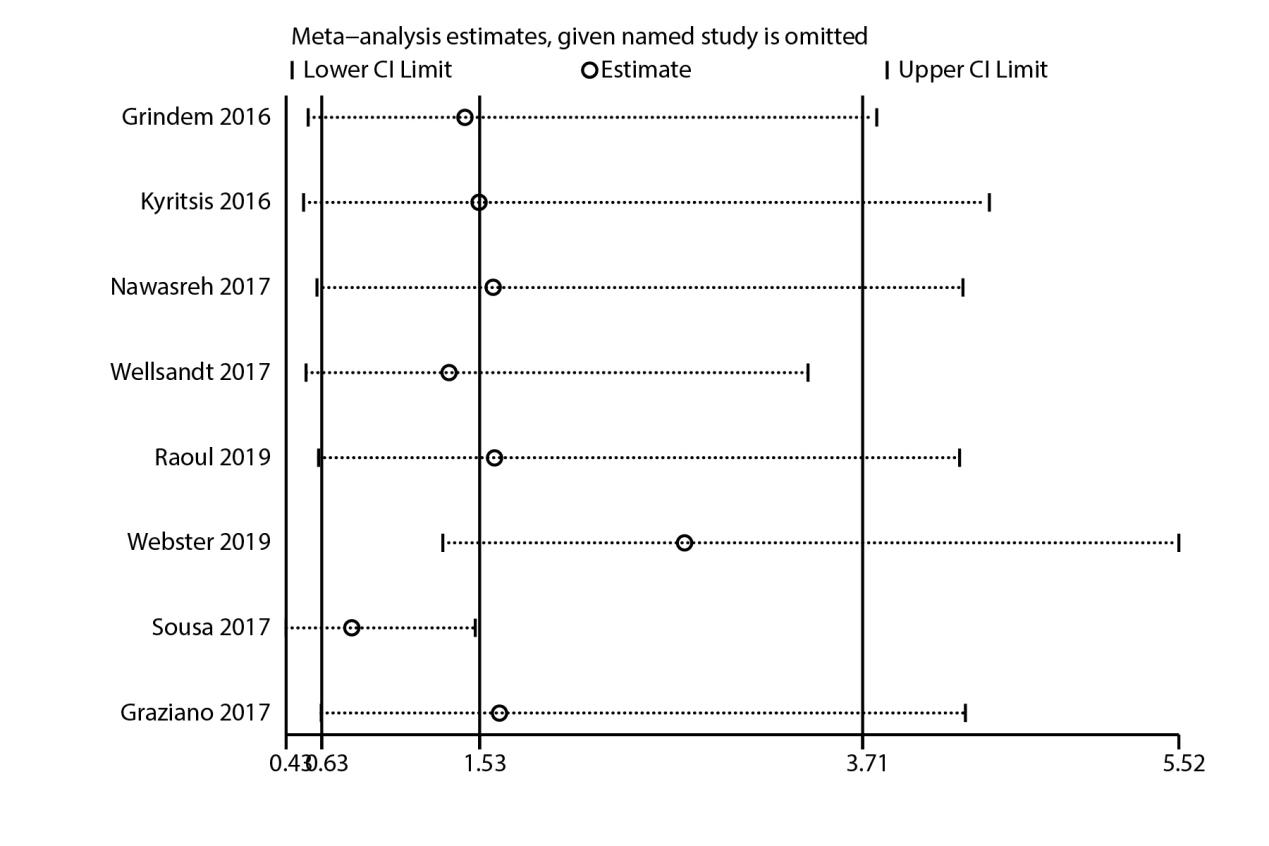


Figure S3. Sensitivity analysis for contralateral ACL injury


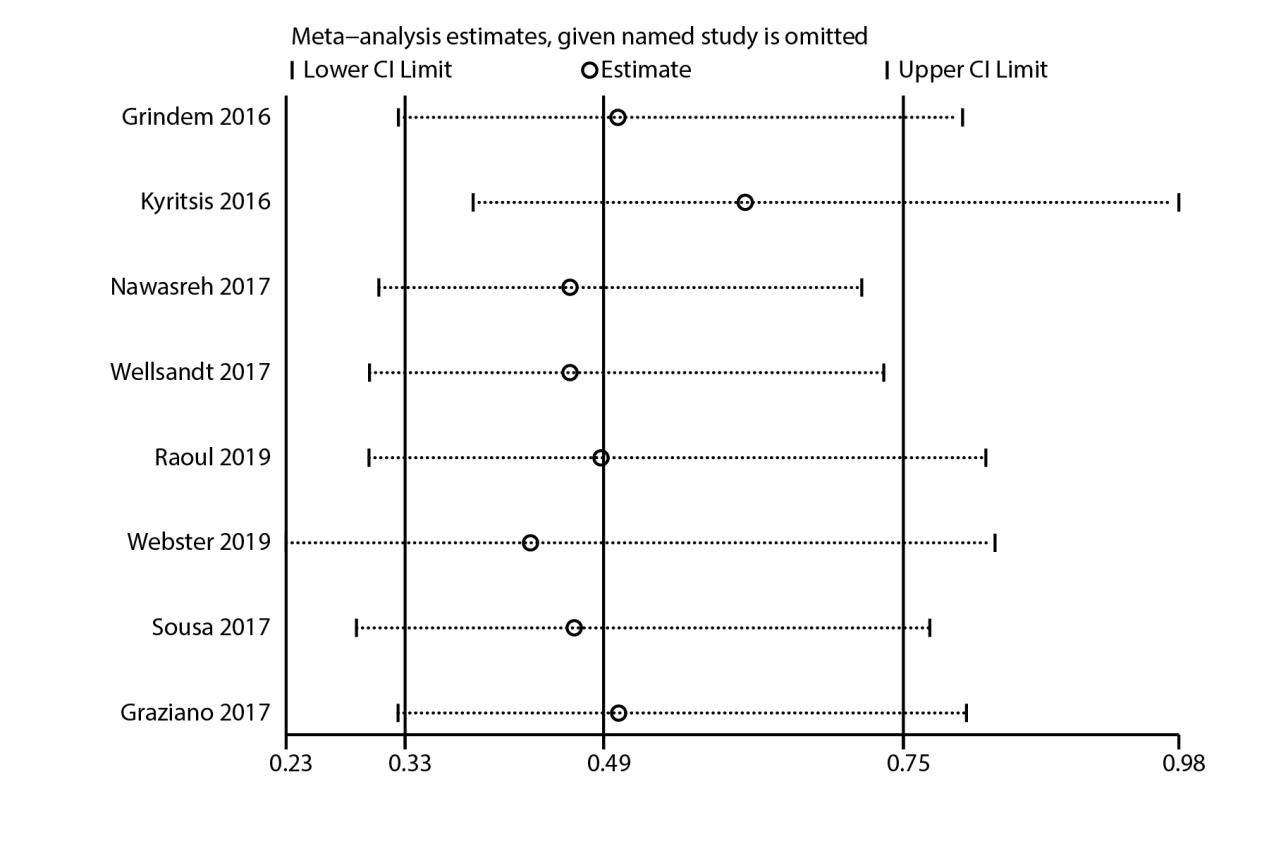


Figure S4. Sensitivity analysis for graft rupture


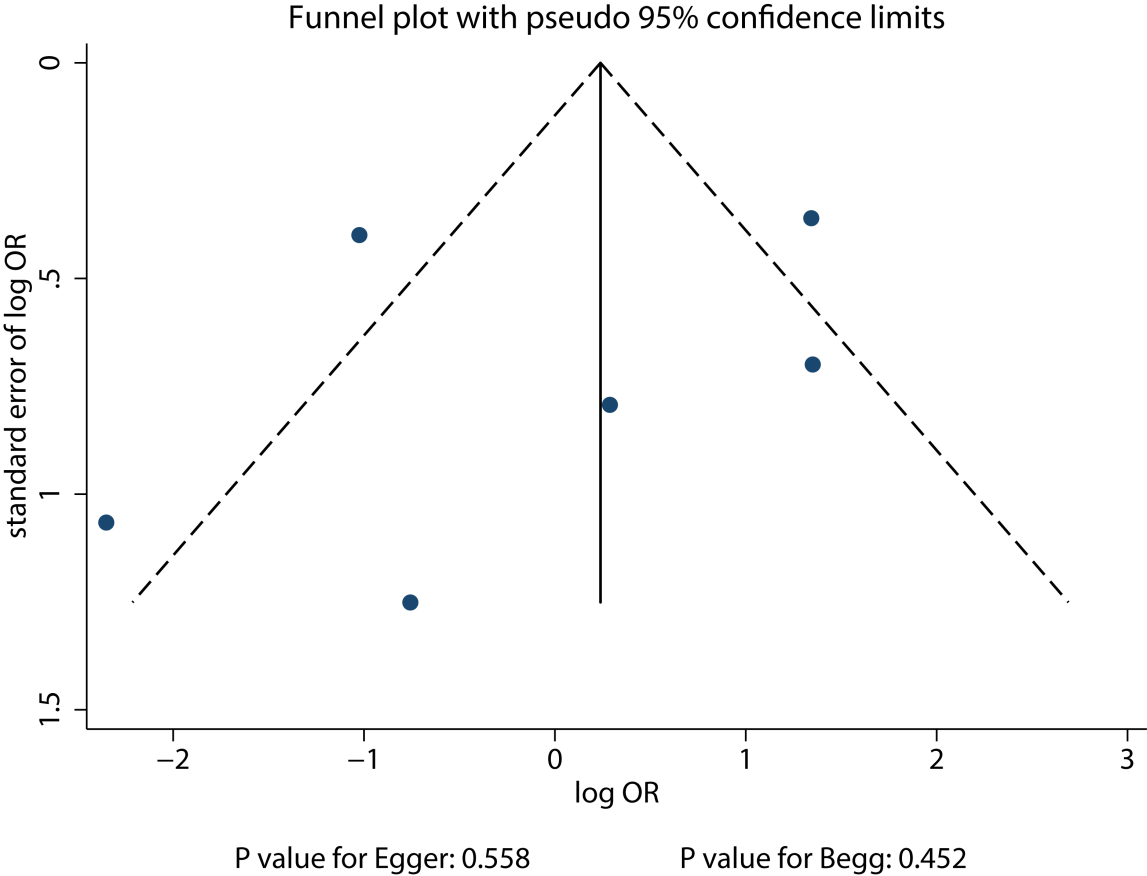


Figure S5. Funnel plot for knee injury


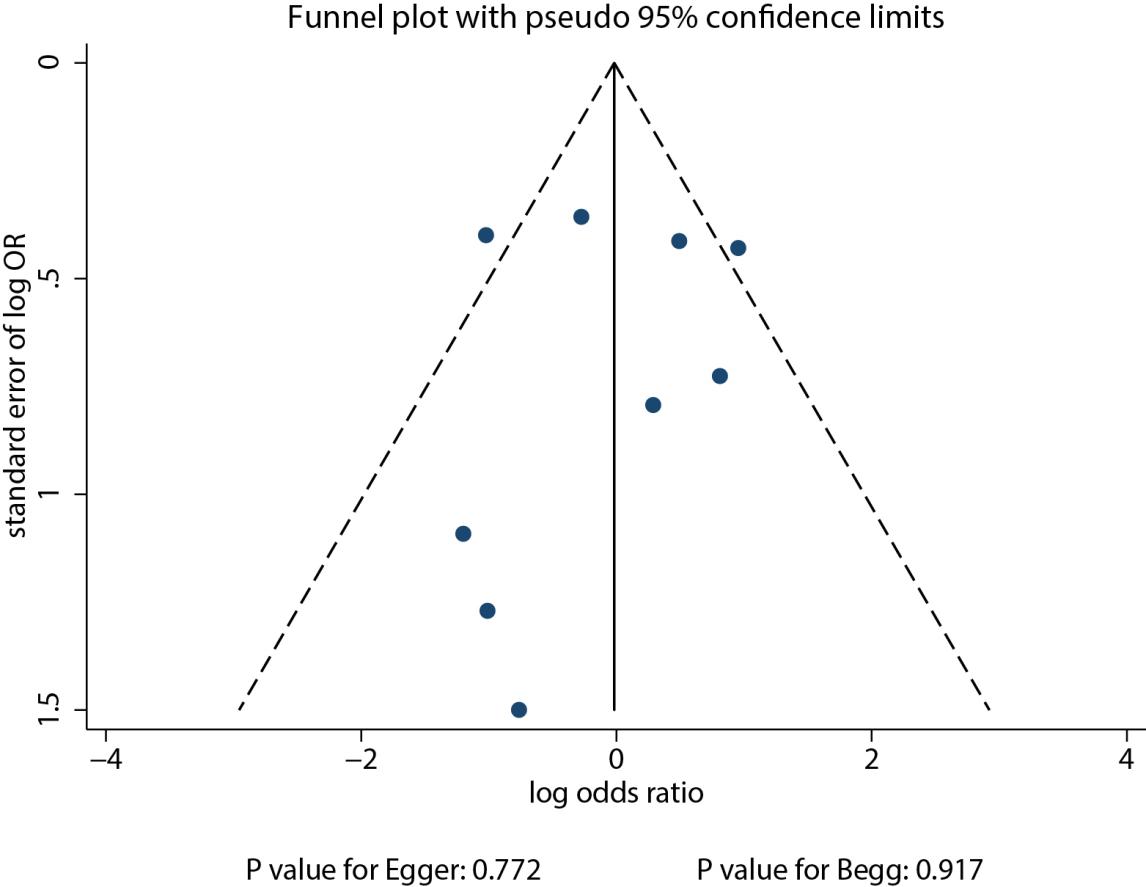


Figure S6. Funnel plot for secondary ACL injury


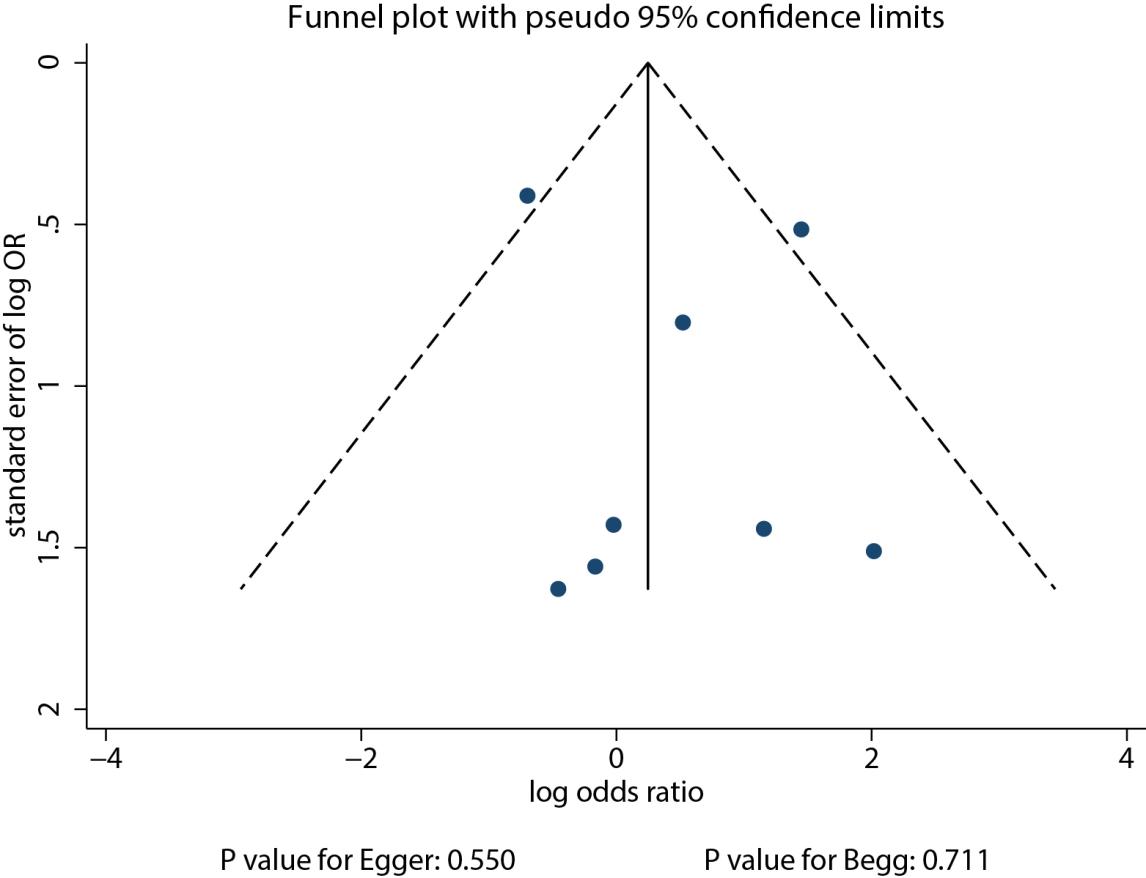


Figure S7. Funnel plot for contralateral ACL injury


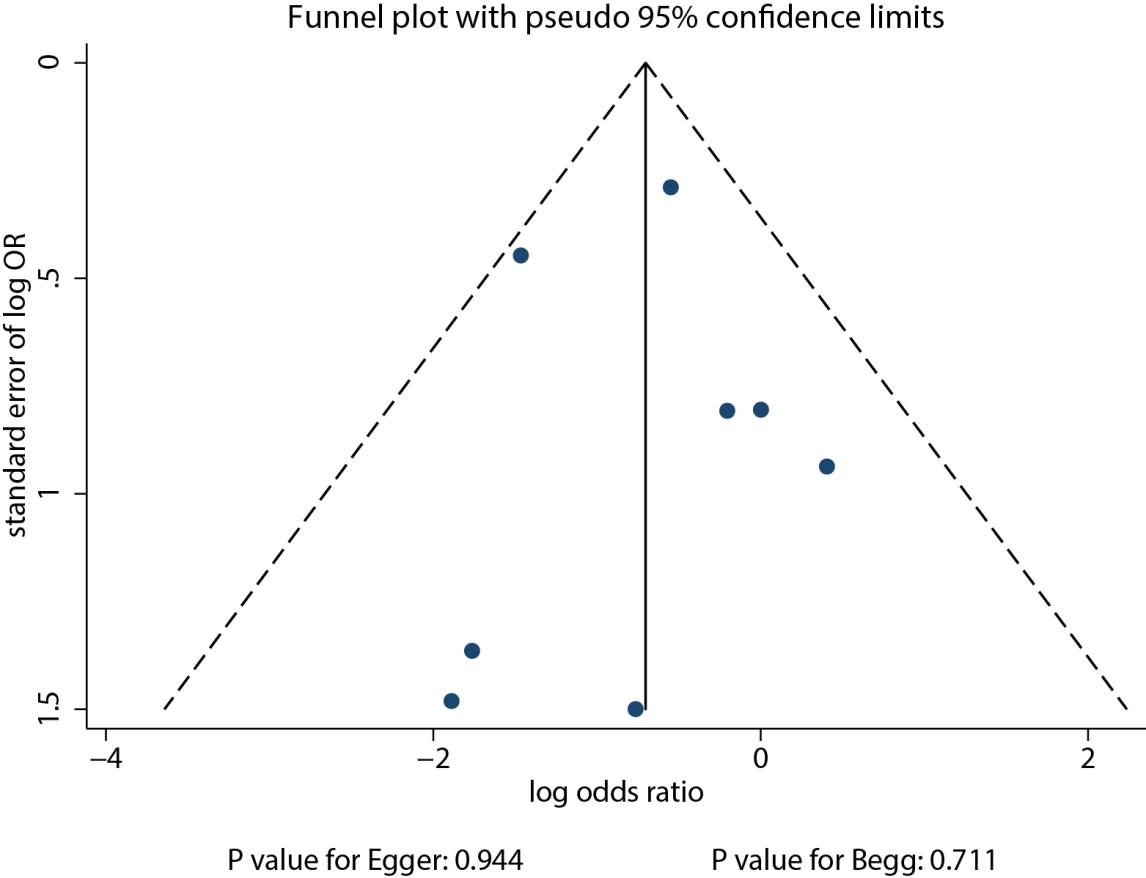


Figure S8. Funnel plot for graft rupture
