# Supplementary material for: Association between passing return-to-sport testing and re-injury risk in patients after anterior cruciate ligament reconstruction surgery: a systematic review and meta-analysis
Source: PeerJ. 2024 Apr 29;12:e17279. doi: 10.7717/peerj.17279 (PMC11064852; doi:10.7717/peerj.17279)
Supplement: Supplemental Information 2 [file peerj-12-17279-s002.docx]

Systematic Review and/or Meta-Analysis Rationale

For systematic reviews / meta-analyses, authors need to provide the following information:

**The rationale for conducting the systematic review / meta-analysis**

Multiple systematic reviews and meta-analyses have already demonstrated the potential significance of the return-to-sport (RTS) test in assessing the prognosis of ligament (ACL) injuries. However, the results of many previous studies are controversial and have the limitation of insufficient follow-up time. Furthermore, there have been additional studies published that should be included in a brand new meta-analysis, necessitating an update of these results. As a result, we undertook a systematic review and meta-analysis to evaluate the association between passing the RTS test and the risk of knee injury, secondary ACL injury, contralateral ACL injury, or graft rupture.

**The contribution that it makes to knowledge in light of previously published related reports, including other meta-analyses and systematic reviews.**

Losciale et al's (1) 4-study analysis found no significant link between passing RTS tests and secondary ACL injury risk. Webster et al's (2) meta-analysis of 17 studies showed equivocal results regarding RTS test battery validity in reducing graft rupture and contralateral ACL injuries. Due to all the controversies and limitations of previous studies, the aim of our study was to conduct a comprehensive analysis of published studies to evaluate the relationship between RTS test and knee injury, secondary ACL injury, contralateral ACL injury, or graft rupture.

**References**

1. Losciale JM, Zdeb RM, Ledbetter L, Reiman MP, Sell TC. The Association Between Passing Return-to-Sport Criteria and Second Anterior Cruciate Ligament Injury Risk: A Systematic Review With Meta-analysis. The Journal of orthopaedic and sports physical therapy. 2019;49(2):43-54.

2. Webster KE, Hewett TE. What is the Evidence for and Validity of Return-to-Sport Testing after Anterior Cruciate Ligament Reconstruction Surgery? A Systematic Review and Meta-Analysis. Sports medicine (Auckland, NZ). 2019;49(6):917-29.
